# Speeding up the core algorithm for the dual calculation of minimal cut sets in large metabolic networks

**DOI:** 10.1186/s12859-020-03837-3

**Published:** 2020-11-09

**Authors:** Steffen Klamt, Radhakrishnan Mahadevan, Axel von Kamp

**Affiliations:** 1 Max Planck Institute for Dynamics of Complex Technical Systems, Sandtorstrasse 1, 39106 Magdeburg, Germany; 2 Department of Chemical Engineering and Applied Chemistry, University of Toronto, 200 College Street, Toronto, ON M5S 3E5 Canada

**Keywords:** Constraint-based modeling, Stoichiometric modeling, Metabolic networks, Metabolic engineering, Computational strain design, Duality, Elementary modes

## Abstract

**Background:**

The concept of minimal cut sets (MCS) has become an important mathematical framework for analyzing and (re)designing metabolic networks. However, the calculation of MCS in genome-scale metabolic models is a complex computational problem. The development of duality-based algorithms in the last years allowed the enumeration of thousands of MCS in genome-scale networks by solving mixed-integer linear problems (MILP). A recent advancement in this field was the introduction of the MCS^2^ approach. In contrast to the Farkas-lemma-based dual system used in earlier studies, the MCS^2^ approach employs a more condensed representation of the dual system based on the nullspace of the stoichiometric matrix, which, due to its reduced dimension, holds promise to further enhance MCS computations.

**Results:**

In this work, we introduce several new variants and modifications of duality-based MCS algorithms and benchmark their effects on the overall performance. As one major result, we generalize the original MCS^2^ approach (which was limited to blocking the operation of certain target reactions) to the most general case of MCS computations with arbitrary target and desired regions. Building upon these developments, we introduce a new MILP variant which allows maximal flexibility in the formulation of MCS problems and fully leverages the reduced size of the nullspace-based dual system. With a comprehensive set of benchmarks, we show that the MILP with the nullspace-based dual system outperforms the MILP with the Farkas-lemma-based dual system speeding up MCS computation with an averaged factor of approximately 2.5. We furthermore present several simplifications in the formulation of constraints, mainly related to binary variables, which further enhance the performance of MCS-related MILP. However, the benchmarks also reveal that some highly condensed formulations of constraints, especially on reversible reactions, may lead to worse behavior when compared to variants with a larger number of (more explicit) constraints and involved variables.

**Conclusions:**

Our results further enhance the algorithmic toolbox for MCS calculations and are of general importance for theoretical developments as well as for practical applications of the MCS framework.

## Background

The computer-aided analysis of complex metabolic networks has become an essential tool to understand functions, properties, and capabilities of the cellular metabolism and to rationally modify it for biotechnological applications. In particular, constraint-based metabolic modeling has evolved as a powerful framework providing a plethora of mathematical techniques to explore genome-scale metabolic networks [1–3]. One particular technique from this toolbox is based on the concept of minimal cut sets (MCS) [4–7]. MCS represent a minimal set of interventions in the metabolism (typically gene or reaction knockouts) that will block a given (target) phenotype. The approach of MCS is very flexible as it allows the user to specify complex phenotypes to be blocked (e.g. growth or phenotypes with low yield of a certain product) and to account for (desired or protected) phenotypes that should be kept feasible when blocking the targeted phenotype. For this reason, and due to a number of useful theoretical properties, the concept of MCS has been used for various applications, for example, to compute synthetic lethals [7, 8], to find targets in cancer cells [8], to identify metabolic engineering strategies (computational strain design) [9–12], or to study the robustness of metabolic networks [13].

The development of algorithms for the efficient calculation of MCS has been a subject of many research activities [6, 7, 14–24]. In the first decade after its inception, MCS were mainly calculated from elementary (flux) modes, which are minimal functional units of metabolic networks that can operate in steady state [25, 26]. Having the complete set of elementary modes (EM) available enables one to fully enumerate all MCS for a given problem [6, 14, 17]. Generally, an MCS problem comprises a metabolic network, the target phenotype(s) to be blocked, and (optionally) desired phenotypes to be protected [6]. Most of the algorithms used to calculate the MCS from the EM are related to the calculation of so-called minimal hitting sets, a method known from hypergraph theory [6, 14, 17]. However, although this approach is straightforward and elegant, it essentially requires the full enumeration of EM in a preprocessing step which is not normally feasible in larger or even genome-scale networks [27]. An important theoretical development to overcome this limitation was achieved by Ballerstein et al. [15] who made use of the Farkas lemma to show that MCS can be calculated as EM in a dual network. Although the full enumeration of MCS (via EM in the dual network) was still infeasible in genome-scale networks, this finding opened a completely new branch of MCS algorithms. In particular, in [7], the duality-based scheme could be employed to conceive a mixed-integer linear program (MILP) that can be used to enumerate the smallest MCS. Importantly, this algorithm now also allowed the calculation of thousands of MCS in genome-scale networks. The fact that the MILP-based algorithm delivers in genome-scale networks only a subset of the MCS with smallest (increasing) cardinality was not limiting since in most applications (especially for strain design) the MCS with the fewest interventions are typically most relevant. Several subsequent studies presented variants and extensions of the duality-based MILP approach [18–21].

A major modification for the duality-based MCS computation was recently proposed by Miraskarshahi et al. [21]. They suggested the use of a more compact dual network based on the nullspace of the stoichiometric matrix reducing the dimension of the problem compared to the Farkas-lemma based dual network derived by Ballerstein et al. [15]. The authors proved the correctness of their dual network approach for MCS calculation (which they named minimal coordinated support MCS = MCS^2^), presented several useful properties of this method and showed that it may speed up the full enumeration of MCS in (smaller) networks whenever this enumeration is feasible. Moreover, motivated by their findings, they also proposed another variant of a duality-based MILP algorithm for the computation of shortest MCS in large networks [21].

In this study we present new theoretical and algorithmic developments that, on the one hand, extend and generalize the results of Miraskarshahi et al. [21], and, on the other hand, may further speed up duality-based MCS algorithms in general. First of all, we noticed that the mentioned new duality approach in [21] may only handle single or multiple target *reactions* but not the more general approach of phenotypes specified by target region(s). Likewise, desired phenotypes cannot be protected which is crucial for applications in metabolic engineering. Furthermore, inhomogeneous constraints (e.g. lower and upper bounds of reaction rates different from zero), which are important features of constraint-based metabolic models, were not considered. We therefore generalize the MCS^2^ approach to allow integration of those specifications. Second, we found that the duality-based MILP algorithm presented by Miraskarshahi et al. [21] did not fully leverage the smaller dual network introduced by the authors and mainly represented a small modification of the traditional MILP formulation [7], which is based on the duality approach of Ballerstein et al. [15]. We therefore develop a new MILP formulation that fully exploits the dimension reduction implied by the nullspace-based dual network representation. We perform several benchmarks and show that our new nullspace-based MILP algorithm is significantly faster than the traditional MILP formulation derived from the Farkas lemma [7]. We finally also present and test different variants of constraints representations in the two MILP formulations and quantify their effect on overall performance. It can be shown that the way constraints are represented in both approaches may have a high impact on algorithm performance and that highly condensed formulations of constraints do not always lead to superior computational performance compared to variants with a larger number of (more explicit) constraints and involved variables.

## Methods

### Definitions

The structure of a metabolic network with $m$ metabolites and $n$ reactions is represented by a stoichiometric matrix ${\mathbf{N}} \in {\mathbb{R}}^{m \times n}$. In steady state with constant concentrations of internal metabolites the vector of reaction rates ${\mathbf{r}} \in {\mathbb{R}}^{n}$ satisfies

1 $$\begin{array}{*{20}c} {\mathbf{Nr} = \mathbf{0}. } \\ \end{array}$$

The reaction rates $r_{i}$ may be constrained by lower $\left( {lb_{i} } \right)$ and upper ($ub_{i}$) bounds expressing, for example, physiological flux limits (e.g. maximal substrate uptake rates or minimal ATP maintenance demand) and irreversibilities ($lb_{i} \ge 0$):

2 $$\begin{array}{*{20}c} {lb_{i} \le r_{i} \le ub_{i} } \\ \end{array}$$

For the sake of simplicity, we assume that $ub_{i} \ge 0$ for all reactions (if this is not the case for a reaction $i$ then its direction can be reversed to fulfill this requirement). Sometimes we will use an equivalent representation of the flux bounds (2):

2a $$\begin{aligned} r_{i} & \le ub_{i} \\ - r_{i} & \le - lb_{i} \\ \end{aligned}$$

By $Irrev = \left\{ {i| lb_{i} \ge 0} \right\}$ we denote the set of indices of irreversible reactions, and with $Rev = \left\{ {i| lb_{i} < 0} \right\}$ the indices of the reversible reactions. Pure reversibility constraints can be expressed by

3 $$r_{i} \ge 0 \enspace \forall i \in Irrev.$$

Other linear constraints on the fluxes, e.g. enzyme allocation constraints [28, 29]*,* can be included in a more general set of linear (in)equalities:

4 $$\begin{array}{*{20}c} {\mathbf{Ar} \le \mathbf{b}.} \\ \end{array}$$

The set of steady-state flux vectors ${\mathbf{r}}$ obeying (1), (2), and (4) span the space of feasible steady-state flux vectors (the flux polyhedron). In some applications and formulations only the steady state (1) and reversibility constraints (3) are considered, in this (homogeneous) case the solution space is the flux cone.

For the calculation of minimal cut sets (MCS), one first needs to specify a target functionality that is to be blocked by the MCS. This can be the operation of a single or a set of *target (or objective) reaction(s)*, i.e., one demands that knocking out the reactions of an MCS implies a zero flux in the target reaction(s) [4, 21]. A more general formulation is based on a *target region* characterized by inequalities posed by a matrix ${\mathbf{T}} \in {\mathbb{R}}^{t \times n}$ and a vector ${\mathbf{t}} \in {\mathbb{R}}^{t}$:

5 $$\begin{array}{*{20}c} {\mathbf{Tr} \le \mathbf{t}} \\ \end{array}$$

Importantly, the target region is spanned by (5) together with (1), (2) and (4) and thus forms a subset (a sub-polyhedron also called target polyhedron) of the flux polyhedron. The case of a target reaction can easily be represented by (5). For example, if $h$ is the index of an irreversible target reaction whose operation is to be blocked by the MCS, then one includes a row in ${\mathbf{T}}$ containing only zeros except a $- 1$ at position $h$ and the corresponding element in ${\mathbf{t}}$ is a negative number $c < 0$. However, for applications in computational strain design one typically needs more complicated formulations going beyond target reactions [e.g., to express (minimum) product yield constraints], which can conveniently be integrated as inequalities in (5). As mentioned above, the target region is defined by (5) together with (1), (2) and (4) and, for technical reasons, we assume in the following that the inhomogeneous flux bounds of (2a) (excluding pure reversibility constraints of type (3); these will be treated separately) as well as the additional constraints (4) have been incorporated in ${\mathbf{T}}$ and ${\mathbf{t}}$; the target region is then spanned by (1), (3) and (5). Again, in applications and formulations that only operate on the flux cone, inhomogeneous (non-zero) flux bounds in (2a) and other constraints of type (4) will not be present. It should also be noted that the target region must not contain the zero vector since otherwise no MCS exists that could block the entire target region (reaction knockouts cannot eliminate the zero flux vector).

A further requirement for many MCS applications is the additional definition of a desired region, which contains the “wanted” stationary flux vectors of which at least some must be kept feasible after introducing the interventions of an MCS. MCS satisfying these constraints (in addition to blocking the target region) are also called *constrained* MCS [6]. However, in the following, we will omit the prefix “constrained” as it should be clear from the context whether constrained MCS are meant or not. As for the target region, the desired region (desired polyhedron) can be specified by suitable inequalities based on a matrix ${\mathbf{D}} \in {\mathbb{R}}^{d \times n}$ and a vector ${\mathbf{d}} \in {\mathbb{R}}^{d}$:

6 $$\begin{array}{*{20}c} \mathbf{Dr} \le \mathbf{d} \\ \end{array}$$

The inequalities (6) may, for example, demand that a minimum growth rate or product yield should be feasible when knocking out the reactions of an MCS. In particular, with appropriate definitions of the target and desired regions one may search for MCS that enforce strong coupling of growth with product synthesis [11, 30], a frequently used strain design principle. Again, the actual desired region is contained in the flux polyhedron of the entire network, i.e., (6) needs to be fulfilled in combination with (1), (2) and (4). Similar as for the target region, we incorporate constraints of type (4) in matrix ${\mathbf{D}}$ and vector ${\mathbf{d}}$, however, the flux bounds (2) are not included in ${\mathbf{D}}$ as they will be treated separately in the optimization problem given below. Accordingly, the desired region is then specified by (1), (2) and (6). Depending on the application, we allow that the desired space can also be empty if the only goal is to block the target region. This case of unconstrained MCS is relevant, for example, when computing synthetic lethals [7, 8].

### Dual network based on Farkas lemma

As already described in the introductory section, for a long time, MCS have been computed from the elementary modes of a given network by variants of the Berge algorithm for computing minimal hitting sets [5, 6, 14, 17]. One disadvantage of this approach is that the EM need to be calculated in a first step before the MCS can be determined. Since EM calculation in genome-scale metabolic networks is usually infeasible [27], this prevented the application of MCS in those cases. The introduction of the dual network approach by Ballerstein et al. [15] was a first major step to overcome this limitation because it allows one to compute MCS directly as EM from a dual network. The dual network introduced in [15] is based on the Farkas lemma and the theory of irreducible inconsistent subsets. Together with small simplifications suggested in [11, 19], the dual network with stoichiometric matrix ${\mathbf{N}}_{dual}$ and dual flux vector ${\mathbf{r}}_{dual}$ reads:

7 $$\begin{aligned} & {\mathbf{N}}_{dual} {\mathbf{r}}_{dual} = \left( {\begin{array}{*{20}c} {{\mathbf{N}}^{{\mathbf{T}}} } & {\mathbf{I}} & { - {\tilde{\mathbf{I}}}_{{{\varvec{Irrev}}}} } & {{\mathbf{T}}^{{\mathbf{T}}} } \\ \end{array} } \right)\left( {\begin{array}{*{20}c} {\mathbf{u}} \\ {\mathbf{v}} \\ {\mathbf{s}} \\ {\mathbf{w}} \\ \end{array} } \right) = \mathbf{0} \\ & {\mathbf{t}}^{{\varvec{T}}} \mathbf{w} \le - c \\ & \mathbf{u} \in {\mathbb{R}}^{m} ,\quad \mathbf{v} \in {\mathbb{R}}^{n} , \quad {v}_{i} \ge 0\quad \forall i \in Irrev, \quad \mathbf{s} \in {\mathbb{R}}_{ \ge 0}^{{\left| {Irrev} \right|}} , \quad \mathbf{w} \in {\mathbb{R}}_{ \ge 0}^{t} , \quad c > 0 \\ \end{aligned}$$

${\mathbf{I}}$ is the $n \times n$ identity matrix and ${\tilde{\mathbf{I}}}_{{{\varvec{Irrev}}}}$ the $n \times \left| {Irrev} \right|$ reduced identity matrix containing one canonical unit vector for each irreversible reaction. The MCS that block all flux vectors of the target region can be obtained as follows: one first computes the EMs ${\mathbf{e}}_{dual}$ of the dual network and discards those dual EM that either violate ${\mathbf{t}}^{{\varvec{T}}} {\mathbf{w}} \le - c$ or are not support-minimal in the ${\mathbf{v}}$-part. The set of MCS is then obtained by taking the support (the active elements) of the ${\mathbf{v}}$-part of the selected EMs. In this formulation, the desired region is not yet considered, however, once the MCS are determined as described above, one may check for each MCS whether at least one vector of the desired region remains feasible after blocking the reactions of the MCS, otherwise the MCS is discarded. The more direct integration of the desired region is discussed below for the MILP-based computation of MCS.

We call the dual network described by (7) the *Farkas-lemma-based (FLB) dual network* and note that its dimension is $n \times \left( {m + n + \left| {Irrev} \right| + t} \right)$. The reactions of the primal have become metabolites in the dual while quite a large number of “reactions” exist in the dual network. The rank of the dual stoichiometric matrix is $n$ and its nullspace [which contains the solutions ${\mathbf{r}}_{dual}$ of (7)] has dimension $m + \left| {Irrev} \right| + t$ (Table 1).

**Table 1:** Comparison of the number of variables and equations and of the dimension of the resulting solution space (nullspace) of the FLB and NB dual system and their corresponding MILP

	FLB dual system [Eq. (7)]	Generalized NB dual system [Eq. (13)]
# dual variables	$m + n + \left| {Irrev} \right| + t$	$n + t$
# (in)equalities	$n + 1$	$n - m + 1 \enspace \enspace$(*)
Dimension of solution space (nullspace) of the dual system	$m + \left| {Irrev} \right| + t$	$m + t$

	FLB MILP [Eq. (14)]	NB MILP [Eq. (16)]
# variables in the corresponding MILP	Continuous: $2n + m + t$	Continuous: $2n + t$
	Binary: $n$	Binary: $n$
# (in)equalities in the corresponding MILP	$n + 1 + m + d$	$\left( {n - m} \right) + 1 + m + d = n + 1 + d$

The number of variables of the MILP includes the variables for the desired system integrated in the MILP, whereas the number of (in)equalities of the MILP excludes (flux) bounds and indicator constraints. $m$: number of metabolites; $n$: number of reactions; $t$: number of rows (inequalities) in matrix ${\mathbf{T}}$/vector ${\mathbf{t}}$ in Eq. (5);${ }d$: number of rows (inequalities) in matrix ${\mathbf{D}}$/vector ${\mathbf{d}};$ $\left| {Irrev} \right|$ number of irreversible reactions. (*) It is assumed that the stoichiometric matrix ${\mathbf{N}}$ has full row rank (conservation relations removed), i.e. rank(${\mathbf{N}}$) = ${ }m$.

### Dual network based on the nullspace

The dimension of the solution space (nullspace) of the FLB dual system appears rather large compared to the nullspace of the primal system which is $n - rank\left( {\mathbf{N}} \right)$ and thus $n - m$ if dependent rows (metabolites) have been removed from ${\mathbf{N}}$ as usually done in standard preprocessing steps (removal of conservation relations) [31]. Indeed, Miraskarshahi et al. [21] showed that a more compact dual network with considerably reduced dimension exists, from which the MCS can be computed as EMs. Their approach, which was designed for the case of target reactions (as a special case of the more general definition of target regions), is based on the following observation. Suppose we are given an irreversible target reaction with index $h$ whose operation in steady state is to be blocked by finding suitable MCS. Suppose there is a metabolite (row) $i$ in ${\mathbf{N}}$ where reaction $h$ produces this metabolite, i.e. $N_{ih} > 0$. Now, a cut set (but not necessarily *minimal* cut set) for target reaction $h$ can be constructed by deleting all reactions that may consume this metabolite, i.e. by deleting all reversible reactions $j$ with $N_{ij} \ne 0$ and all irreversible reactions $j$ with $N_{ij} < 0$. This set

8 $$R\left( {{\mathbf{N}}_{i, \cdot } } \right) = \{ j|N_{ij} < 0\} \cup \left\{ {j\left| {j \in Rev \wedge N_{ij}>0 } \right.} \right\}$$

is called the *coordinated support* of the (metabolite row) vector ${\mathbf{N}}_{i, \cdot }$ and inactivating these reactions ensures that the target reaction cannot operate anymore because it would otherwise violate the steady-state condition (1). Miraskarshahi et al. [21] next concluded that this relationship holds true for any artificial metabolite ${\mathbf{p}}$ obtained by linear combinations of rows (metabolites) of the stoichiometric matrix (${\mathbf{p}} = {\mathbf{N}}^{{\mathbf{T}}} {\mathbf{g}}$) and showed that such linear combinations are in fact sufficient to enumerate all MCS. Hence, the goal is to find linear combinations of the metabolites (i.e., a suitable vector of the row space of ${\mathbf{N}}$) such that the coordinated support of the resulting virtual metabolite becomes minimal. An elegant solution to find these vectors was also suggested in [21]: one computes a kernel matrix ${\mathbf{K}}$, which contains a basis of the nullspace of the stoichiometric matrix ${\mathbf{N}}$ (i.e. ${\mathbf{NK}} = 0)$, and considers its transpose ${\mathbf{K}}^{{\mathbf{T}}}$ as stoichiometric matrix of the dual network together with corresponding steady-state conditions for the dual metabolites:

9 $$\begin{aligned} & {\mathbf{N}}_{dual} {\mathbf{r}}_{dual} = {\mathbf{K}}^{{\mathbf{T}}} {\mathbf{r}}_{dual} = \mathbf{0} \\ & {\mathbf{r}}_{dual} \in {\mathbb{R}}^{n} \\ \end{aligned}$$

Due to the construction, the reactions in this dual network are in 1:1 correspondence to the reactions of the original (primal) network but in the dual they are all treated as reversible. The metabolites of the dual system represent the degrees of freedoms of the primal system. From linear algebra it is known that the nullspace of the nullspace yields the primal row space, here of ${\mathbf{N}}$. The trick is now to compute the EM of the dual network which delivers all support-minimal solutions of the row-space of ${\mathbf{N}}$. Since all reactions in the dual system (9) are treated as reversible, each resulting EM of the dual, ${\mathbf{e}}_{dual}$, is reversible, i.e., $- {\mathbf{e}}_{dual}$ also solves (9). After computation, we select all EM where the target reaction is involved and assume that the target reaction is positive (otherwise the EM can be multiplied by $- \,1$). From these dual EM one may identify the ones with minimal coordinated support with respect to the target reaction which are then the MCS. Note that the reversibility of the reactions in the primal is thus accounted for by the coordinated support (8), which differentiates between reversible and irreversible reactions. Although the correctness of the MCS^2^ approach was also proven by means of the Farkas lemma [21], we call (9) the nullspace-based (NB) dual system to contrast it with the FLB dual system (7).

We note that the dimension of the NB dual network is $\left( {n - rank\left( {\mathbf{N}} \right)} \right) \times n$ and thus $\left( {n - m} \right) \times n$ if we again assume full row rank of ${\mathbf{N}}$. Accordingly, compared to the FLB dual network (7), even if we neglect the dimension of matrix ${\mathbf{T}}$, which allows specification of more complex target regions, there are $m + \left| {Irrev} \right|$ less dual reactions (columns in ${\mathbf{N}}_{dual}$) and $m$ less dual metabolites (rows) showing that this representation is indeed more compact. Furthermore, the NB dual system has only $m$ degrees of freedom (dimension of its nullspace) which is also reduced compared to the FLB system. The NB approach requires the determination of the kernel (nullspace) matrix of ${\mathbf{N}}$, which, however, has low computational costs. As mentioned by the authors in [21], another advantage of the MCS^2^ approach is that the EM of the dual network can be used to determine the MCS for any target reaction or any combination of target reactions, hence, a recalculation of the EM is not necessary if the target reaction is changed. However, the NB approach in its current formulation cannot be applied with arbitrary target regions with inhomogeneous (non-zero) flux bounds or with more general constraints of type (4), moreover, it cannot deal with desired fluxes to be protected and is thus limited to a smaller class of applications. In the following we modify the NB dual network approach to allow incorporation of target and later of desired regions.

### Incorporating arbitrary target regions in the NB dual network

We recall that a target region is specified by (1), (3) and (5)

$$\begin{aligned} & \mathbf{Nr} = \mathbf{0} \\ & r_{i} \ge 0 \enspace \forall i \in Irrev \\ & \mathbf{Tr} \le \mathbf{t} \\ \end{aligned}$$

and that the network’s inhomogeneous flux bounds (2a) and other constraints of type (4) have been integrated in ${\mathbf{T}}$ and ${\mathbf{t}}$, respectively. By introducing slack variables ${\mathbf{w}}$, this inhomogeneous system of linear inequalities can be recast to an extended homogeneous system of linear equations with one remaining single inequality:

10 $$\begin{aligned} & {\mathbf{N}}_{{\varvec{e}}} {\mathbf{r}}_{{\varvec{e}}} = \left( {\begin{array}{*{20}c} {\mathbf{N}} & \mathbf{0} & \mathbf{0} \\ {\mathbf{T}} & {\mathbf{I}} & { - {\mathbf{t}}} \\ \end{array} } \right)\left( {\begin{array}{*{20}c} {\mathbf{v}} \\ {\mathbf{w}} \\ y \\ \end{array} } \right) = \mathbf{0} \\ & \mathbf{v} \in {\mathbb{R}}^{n} , \quad {v}_{i} \ge 0 \quad \forall i \in Irrev, \quad \mathbf{w} \in {\mathbb{R}}_{ \ge 0}^{t} , \quad y = 1 \\ \end{aligned}$$

Here, ${\mathbf{I}}$ is the $t \times t$ identity matrix. Putting the $y = 1$ inequality aside for a moment, we can now apply the NB dual network approach (9). For this we need a kernel matrix ${\mathbf{K}}_{{\varvec{e}}}$ for the extended system (10) which can be easily constructed as

11 $$\begin{array}{*{20}c} {{\mathbf{K}}_{{\varvec{e}}} = \left( {\begin{array}{*{20}c} {\mathbf{K}} & \mathbf{0} \\ { - {\mathbf{TK}}} & {\mathbf{t}} \\ \mathbf{0} & 1 \\ \end{array} } \right),} \\ \end{array}$$

where ${\mathbf{K}}$ is, as before, the kernel matrix of the stoichiometric matrix ${\mathbf{N}}$ of the original system. For the NB dual network (9) of the extended system (10) we thus obtain:

12 $$\begin{array}{*{20}c} {{\mathbf{N}}_{{{\varvec{e}},dual}} {\mathbf{r}}_{{{\varvec{e}},dual}} = {\mathbf{K}}_{{\varvec{e}}}^{{\mathbf{T}}} {\mathbf{r}}_{{{\varvec{e}},dual}} = \left( {\begin{array}{*{20}c} {{\mathbf{K}}^{{\mathbf{T}}} } & { - {\mathbf{K}}^{{\mathbf{T}}} {\mathbf{T}}^{{\mathbf{T}}} } & \mathbf{0} \\ \mathbf{0} & {{\mathbf{t}}^{{\mathbf{T}}} } & 1 \\ \end{array} } \right)\left( {\begin{array}{*{20}c} {\mathbf{v}} \\ {\mathbf{w}} \\ y \\ \end{array} } \right) = \mathbf{0}} \\ \end{array}$$

We still need to take into account that $y = 1$ and from (12) we see that this holds if and only if ${\mathbf{t}}^{{\varvec{T}}} {\mathbf{w}} = - 1$. We can thus also write

13 $$\begin{aligned} & {\mathbf{N}}_{{{\varvec{e}},dual}} {\mathbf{r}}_{{{\varvec{e}},dual}} = {\mathbf{K}}_{{\varvec{e}}}^{{\mathbf{T}}} {\mathbf{r}}_{{{\varvec{e}},dual}} = \left( {\begin{array}{*{20}c} {{\mathbf{K}}^{{\mathbf{T}}} } & { - {\mathbf{K}}^{{\mathbf{T}}} {\mathbf{T}}^{{\mathbf{T}}} } \\ \end{array} } \right)\left( {\begin{array}{*{20}c} {\mathbf{v}} \\ {\mathbf{w}} \\ \end{array} } \right) = \mathbf{0} \\ & {\mathbf{t}}^{{\varvec{T}}} \mathbf{w} = - 1 \\ & \mathbf{v} \in {\mathbb{R}}^{n} , \quad \mathbf{w} \in {\mathbb{R}}_{ \ge 0}^{t} \\ \end{aligned}$$

Note that constraint ${\mathbf{t}}^{{\varvec{T}}} {\mathbf{w}} = - 1$ can be replaced with ${\mathbf{t}}^{{\varvec{T}}} {\mathbf{w}} \le - c, c > 0$ [which is equivalent to the related constraint in Eq. (7)] or simply with ${\mathbf{t}}^{{\varvec{T}}} {\mathbf{w}} < 0$, because the signed support of the solution $$\left( {\begin{array}{*{20}c} {\mathbf{v}} \\ {\mathbf{w}} \\ \end{array} } \right)$$ is identical for any $c > 0$ implying identical MCS. To apply the MCS^2^ approach for this generalized system (13), we first compute the EM for (13) and discard all EM for which ${\mathbf{t}}^{{\varvec{T}}} {\mathbf{w}} < 0$ does not hold. We then drop the ${\mathbf{w}}$-part of the remaining EM vectors and determine their coordinated support $R$ [Eq. (8)]. The set of minimal coordinated supports finally represents the set of MCS. As explained for the FLB dual system, if also desired regions have been defined for the MCS problem, for all obtained MCS it can be easily tested which of them keep some flux vectors feasible in the desired region, all others are discarded.

We note that the dimension of the dual stoichiometric matrix in (13) is $\left( {n - m} \right) \times \left( {n + t} \right)$ which is significantly smaller than the FLB dual network (7). A comparison of the dimensions of the FLB versus the (generalized) NB dual system and of the associated solution spaces is given in Table 1. For the simple case of target reactions [treatable with the original NB approach (9)], system (13) is only slightly larger than (9) as it adds only one additional row in ${\mathbf{T}}$ [and thus one additional column in ${\mathbf{N}}_{{{\varvec{e}},dual}}$ in (13)] for each target reaction. On the other hand, a filtering of all EM for the participation of the target reaction(s) (as needed by the original MCS^2^ approach) is then not necessary anymore.

### Computing MCS from the FLB dual system via a mixed-integer linear program

While both the FLB and the NB dual networks allow direct enumeration of MCS as EM of a dual system, they are in this form still limited to smaller networks since full enumeration of MCS (EM in the dual system) remains infeasible in genome-scale networks. The development of duality-based MCS calculation schemes was nevertheless valuable as they provide a framework for the optimization-based calculation of the shortest MCS (MCS with smallest cardinality), which are most relevant in realistic applications [7]. Starting with the FLB dual network (7), searching for the smallest MCS translates to the task to find a vector $${\mathbf{r}}_{dual} = \left( {\begin{array}{*{20}c} {\mathbf{u}} \\ {\mathbf{v}} \\ {\mathbf{s}} \\ {\mathbf{w}} \\ \end{array} } \right)$$ which has minimal support in ${\mathbf{v}}$. This optimization problem requires the introduction of integer variables indicating the support of ${\mathbf{v}}$, i.e. the deleted reactions, and the optimization problem thus becomes a mixed-integer linear program (MILP). Since a MILP can deal with any type of linear (in)equalities and does not need a zero on the right-hand side in (7), structure and dimension of the system can partially be simplified. Moreover, the requirement that vectors of the desired system [posed by constraints (1), (2) and (6)] must be feasible can also directly be integrated in the MILP. There have been different MILP variants in treating the $n \times n$ identity matrix ${\mathbf{I}}$ and its associated vector ${\mathbf{v}}$ in (7), for example, they can be split into separate parts for reversible and irreversible reactions [11]. The arguably most compact representation of the MILP based on the FLB dual network reads as follows:

14 $$\begin{aligned} & minimize \mathop \sum \limits_{i} z_{i} \\ & s.t. \\ & \\ & \left( {\begin{array}{*{20}c} {{\mathbf{N}}^{{\mathbf{T}}} } & {\mathbf{I}} & {{\mathbf{T}}^{{\mathbf{T}}} } & \mathbf{0} \\ \mathbf{0} & \mathbf{0} & {{\mathbf{t}}^{{\mathbf{T}}} } & \mathbf{0} \\ \mathbf{0} & \mathbf{0} & \mathbf{0} & {\mathbf{N}} \\ \mathbf{0} & \mathbf{0} & \mathbf{0} & {\mathbf{D}} \\ \end{array} } \right)\left( {\begin{array}{*{20}c} {\mathbf{u}} \\ {\mathbf{v}} \\ {\mathbf{w}} \\ {\mathbf{r}} \\ \end{array} } \right)\begin{array}{*{20}c} = \\ \le \\ = \\ \le \\ \end{array} \left( {\begin{array}{*{20}c} \mathbf{0} \\ { - c} \\ \mathbf{0} \\ {\mathbf{d}} \\ \end{array} } \right) \\ & \\ & \forall i \in Irrev: z_{i} = 0 \to v_{i} \le 0 \\ & \forall i \in Rev:z_{i} = 0 \to v_{i} = 0 \\ & \left( {1 - z_{i} } \right) \cdot lb_{i} \le r_{i} \le \left( {1 - z_{i} } \right) \cdot ub_{i} \\ & {\mathbf{u}} \in {\mathbb{R}}^{m} ,{ }\quad {\mathbf{t}} \in {\mathbb{R}}^{t} ,{ }\quad {\mathbf{w}} \in {\mathbb{R}}_{ \ge 0}^{t} ,{ }\quad {\mathbf{v}},{\mathbf{r}} \in {\mathbb{R}}^{{n}} ,{ }\quad c > 0,{ }\quad z_{i} \in \left\{ {0,1} \right\} \\ \end{aligned}$$

Blocking of the target region is demanded via the first two rows in the matrix which express the duality-based relationship (7). The associated variables for this dual subsystem are ${\mathbf{u}}$, ${\mathbf{v}}$ and ${\mathbf{w}}$. In contrast to the target region, the requirement of feasible flux vectors in the desired region (third and fourth row in the matrix in Eq. (14) as well as the flux bounds for the associated flux variable ${\mathbf{r}}$) are formulated via their primal representation [cf. Eqs. (1), (2) and (6)]. The continuous dual (${\mathbf{u}},{\mathbf{v}},{\mathbf{w}}$) and primal (${\mathbf{r}}$) variables are interconnected via the binary $z_{i}$ variables. The latter are directly linked to the dual ${\mathbf{v}}$ variables via indicator constraints. The $z_{i}$ being 1 mark the cuts, which happens if a reversible reaction $i$ has a non-zero $v_{i}$ or if an irreversible reaction $i$ has a positive $v_{i}$ (hence, negative entries for irreversible reactions in ${\mathbf{v}}{ }$ do not count). The smallest MCS can be found by the given objective function which minimizes the number of cuts.

Note that the constraints for the indicator variables for irreversible reactions as used above incorporate the original constraints expressed by the $\widetilde{{ - {\mathbf{I}}}}_{{{\varvec{Irrev}}}}$ submatrix and its associated variables ${\mathbf{s}}$ in (7), which thus become obsolete. The indicator constraints in (14) have been reduced and simplified compared to previous versions. In particular, each dual reaction variable $v_{i}$ has only one associated indicator variable and no explicit constraint has been specified for the case $z_{i} = 1$. In principle, in Eq. (14), $z_{i}$ could be 1 (and thus indicate a cut) even if $v_{i} = 0$. However, with the objective function minimizing the number of cuts the solver will always seek to set all $z_{i}$ to zero as long as the indicator constraint for $z_{i} = 0$ in (14) allows that. A similar simplification was used in [21].

As already mentioned above there are variants in treating the identity matrix ${\mathbf{I}}$ and the associated dual reaction vector ${\mathbf{v}}$ and its associated Boolean variables $z_{i}$. In particular, the $v_{i}$ entries of reversible and irreversible reactions have often been separated and then been split for reversible reactions [7, 11, 20]. Using equivalent (reduced) indicator constraints as in (14), the split variant, which we will later compare with the more compact version (14), reads as follows:

14a $$\begin{aligned} & minimize \mathop \sum \limits_{i} z_{i} \\ & s.t. \\ & \left( {\begin{array}{*{20}c} {{\mathbf{N}}_{{{\mathbf{Irrev}}}}^{{\mathbf{T}}} } & {{\mathbf{I}}_{{{\mathbf{Irrev}}}} } & \mathbf{0} & \mathbf{0} & {{\mathbf{T}}_{{{\mathbf{Irrev}}}}^{{\mathbf{T}}} } & \mathbf{0} \\ {{\mathbf{N}}_{{{\mathbf{Rev}}}}^{{\mathbf{T}}} } & \mathbf{0} & {{\mathbf{I}}_{{{\mathbf{Rev}}}} } & { - {\mathbf{I}}_{{{\mathbf{Rev}}}} } & {{\mathbf{T}}_{{{\mathbf{Rev}}}}^{{\mathbf{T}}} } & \mathbf{0} \\ \mathbf{0} & \mathbf{0} & \mathbf{0} & \mathbf{0} & {{\mathbf{t}}^{{\mathbf{T}}} } & \mathbf{0} \\ \mathbf{0} & \mathbf{0} & \mathbf{0} & \mathbf{0} & \mathbf{0} & {\mathbf{N}} \\ \mathbf{0} & \mathbf{0} & \mathbf{0} & \mathbf{0} & \mathbf{0} & {\mathbf{D}} \\ \end{array} } \right)\left( {\begin{array}{*{20}c} {\mathbf{u}} \\ {{\mathbf{v}}_{{{\mathbf{Irrev}}}} } \\ {{\mathbf{v}}_{{{\mathbf{Rev}}}}^{{\mathbf{p}}} } \\ {{\mathbf{v}}_{{{\mathbf{Rev}}}}^{{\mathbf{n}}} } \\ {\mathbf{w}} \\ {\mathbf{r}} \\ \end{array} } \right)\begin{array}{*{20}c} \ge \\ = \\ \le \\ = \\ \le \\ \end{array} \left( {\begin{array}{*{20}c} \mathbf{0} \\ \mathbf{0} \\ { - c} \\ \mathbf{0} \\ {\mathbf{d}} \\ \end{array} } \right) \\ & \forall i \in Irrev: z_{i} = 0 \to v_{Irrev, i} = 0 \\ & \forall i \in Rev: z_{i} = 0 \to v_{Rev,i}^{p} = 0 \wedge v_{Rev,i}^{n} = 0 \\ & \left( {1 - z_{i} } \right) \cdot lb_{i} \le r_{i} \le \left( {1 - z_{i} } \right) \cdot ub_{i} \\ & \mathbf{u} \in {\mathbb{R}}^{m},\quad { }{\mathbf{t}} \in {\mathbb{R}}^{t} ,\quad {\mathbf{w}} \in {\mathbb{R}}_{ \ge 0}^{t} ,\quad {\mathbf{v}}_{{{\mathbf{Irrev}}}} \in {\mathbb{R}}_{ \ge 0}^{{\left| {Irrev} \right|}} ,\\ & {\mathbf{v}}_{{{\mathbf{Rev}}}}^{{\mathbf{p}}} , {\mathbf{v}}_{{{\mathbf{Rev}}}}^{{\mathbf{n}}} \in {\mathbb{R}}_{ \ge 0}^{{\left| {Rev} \right|}} , \quad {\mathbf{r}} \in {\mathbb{R}}^{n} ,{ }\quad c > 0,\quad { }z_{i} \in \left\{ {0,1} \right\} \\ \end{aligned}$$

The subscripts ${\mathbf{Irrev}}$ and ${\mathbf{Rev}}$ at the matrices ${\mathbf{N}}^{{\mathbf{T}}}$ and ${\mathbf{T}}^{{\mathbf{T}}} \user2{ }$ indicate the respective submatrices containing the columns associated with the irreversible and reversible reactions, respectively. In (14a), the identity matrix and the ${\mathbf{v}}$ vector from (14) have accordingly been separated in two parts associated with irreversible and reversible reactions, and the reversible part has in turn be split into a positive and a negative part. (Note: with slight abuse of notation, in the term $\forall i \in Irrev: z_{i} = 0 \to v_{Irrev, i} = 0$, the variable $v_{Irrev, i}$ refers to the associated value of the irreversible reaction with original index $i$ in the new vector ${\mathbf{v}}_{{{\mathbf{Irrev}}}}$, the same interpretation holds for the indicator constraints of the reversible reactions.) Instead of allowing a $v_{Irrev, i}$ to become negative in the absence of a cut, which then effectively turns it into a slack variable, we now use ≥ constraints for the first $\left| {Irrev} \right|$ rows associated with irreversible reactions. This formulation requires $\left| {Rev} \right|$ more continuous and $\left| {Rev} \right|$ more Boolean variables but now all ${\mathbf{v}}$ variables are constrained to be non-negative which simplifies the indicator constraints.

The MCS $C$ computed by (14) or (14a) is given by $C = \{ i|z_{i} = 1\}$. Multiple MCS solutions (with increasing cardinality) can be found by adding integer cut constraints to the MILP for a previously found solution. If $C_{k}$ denotes the set of reaction indices that are knocked-out in the *k*-th MCS, then the integer cut constraint are given by

15 $$\begin{array}{*{20}c} {\mathop \sum \limits_{{j \in C_{k} }} z_{j} \le \left| {C_{k} } \right| - 1.} \\ \end{array}$$

In practice, we successively enumerate MCS with increasing size following the procedure introduced in [7]: during the $q$-th iteration of this procedure all MCS of size $q$ are being enumerated using the populate feature of CPLEX. At the end of each iteration exclusion constraints (15) for the newly found MCS are added to prevent supersets of them being found as solutions in the next iteration.

### Computing MCS from the NB dual system via MILP

Together with their NB-based dual system and the MCS^2^ method, Miraskarshahi et al. [21] presented also a MILP for the calculation of shortest MCS for a given set of target reactions. As central constraint for the continuous variables they derived ${\mathbf{u}}^{{\mathbf{T}}} {\mathbf{N}} = {\mathbf{v}}$. However, with ${\mathbf{u}}$ being not sign-limited, this can be rewritten to $$\left( {\begin{array}{*{20}c} {{\mathbf{N}}^{{\mathbf{T}}} } & {\mathbf{I}} \\ \end{array} } \right)\left( {\begin{array}{*{20}c} {\mathbf{u}} \\ {\mathbf{v}} \\ \end{array} } \right) = 0$$ which is the core of (14), hence, this approach represents merely another variant of the MILP based on the FLB dual system (without desired/target regions). In fact, the MILP they presented does not exploit the reduced dimensionality of the NB dual network. Taking the derived NB dual system for arbitrary target regions (13) as starting point and using similar indicator constraints and an analogous integration of the desired system as in (14), a suitable MILP for computing the shortest MCS via the NB dual network can be constructed as follows:

16 $$\begin{aligned} & minimize \mathop \sum \limits_{i} z_{i} \\ & s.t. \\ & \\ & \left( {\begin{array}{*{20}c} {{\mathbf{K}}^{{\mathbf{T}}} } & {{\mathbf{K}}^{{\mathbf{T}}} \cdot {\mathbf{T}}^{{\mathbf{T}}} } & \mathbf{0} \\ \mathbf{0} & {{\mathbf{t}}^{{\mathbf{T}}} } & \mathbf{0} \\ \mathbf{0} & \mathbf{0} & {\mathbf{N}} \\ \mathbf{0} & \mathbf{0} & {\mathbf{D}} \\ \end{array} } \right)\left( {\begin{array}{*{20}c} {\mathbf{v}} \\ {\mathbf{w}} \\ {\mathbf{r}} \\ \end{array} } \right)\begin{array}{*{20}c} = \\ \le \\ = \\ \le \\ \end{array} \left( {\begin{array}{*{20}c} \mathbf{0} \\ { - c} \\ \mathbf{0} \\ {\mathbf{d}} \\ \end{array} } \right) \\ & \\ & \forall i \in Irrev: z_{i} = 0 \to v_{i} \le 0 \\ & \forall i \in Rev: z_{i} = 0 \to v_{i} = 0 \\ & \left( {1 - z_{i} } \right) \cdot lb_{i} \le r_{i} \le \left( {1 - z_{i} } \right) \cdot ub_{i} \\ { } & {\mathbf{t}} \in {\mathbb{R}}^{t} ,{ }\quad {\mathbf{w}} \in {\mathbb{R}}_{ \ge 0}^{t} ,\quad { }c > 0,{ }\quad { }{\mathbf{v}}, {\mathbf{r}} \in {\mathbb{R}}^{{\varvec{n}}} ,\quad { }z_{i} \in \left\{ {0,1} \right\} \\ \end{aligned}$$

As for the two different dual networks, this MILP has also reduced dimensions and there-fore less variables compared to the FLB MILP (14) (see Table 1). A closer inspection also reveals a fundamental relationship between the two formulations (14) and (16): the FLB MILP (14) can directly be converted to the NB MILP (16) by multiplying the first row of the matrix with ${\mathbf{K}}^{{\mathbf{T}}}$. Since ${\mathbf{K}}^{{\mathbf{T}}} {\mathbf{N}}^{{\mathbf{T}}} = {\mathbf{NK}} = \mathbf{0}$, the first column in the matrix in (14) completely vanishes and can be removed yielding system (16).

Analogous to the FLB MILP (14a), an alternative formulation of the NB MILP (16) can be obtained by separating the $v_{i}$ entries of irreversible and reversible reactions and subsequent splitting of $v_{i}$ for reversible reactions. We will later compare this variant with the most condensed version (16):

16a $$\begin{aligned} & minimize \mathop \sum \limits_{i} z_{i} \\ & s.t. \\ & \left( {\begin{array}{*{20}c} {{\mathbf{K}}_{{{\mathbf{Irrev}}}}^{{\mathbf{T}}} } & {{\mathbf{K}}_{{{\mathbf{Rev}}}}^{{\mathbf{T}}} } & { - {\mathbf{K}}_{{{\mathbf{Rev}}}}^{{\mathbf{T}}} } & {{\mathbf{K}}^{{\mathbf{T}}} \cdot {\mathbf{T}}^{{\mathbf{T}}} } & \mathbf{0} \\ \mathbf{0} & \mathbf{0} & \mathbf{0} & {{\mathbf{t}}^{{\mathbf{T}}} } & \mathbf{0} \\ \mathbf{0} & \mathbf{0} & \mathbf{0} & \mathbf{0} & {\mathbf{N}} \\ \mathbf{0} & \mathbf{0} & \mathbf{0} & \mathbf{0} & {\mathbf{D}} \\ \end{array} } \right)\left( {\begin{array}{*{20}c} {{\mathbf{v}}_{{{\mathbf{Irrev}}}} } \\ {{\mathbf{v}}_{{{\mathbf{Rev}}}}^{{\mathbf{p}}} } \\ {{\mathbf{v}}_{{{\mathbf{Rev}}}}^{{\mathbf{n}}} } \\ {\mathbf{w}} \\ {\mathbf{r}} \\ \end{array} } \right)\begin{array}{*{20}c} = \\ \le \\ = \\ \le \\ \end{array} \left( {\begin{array}{*{20}c} \mathbf{0} \\ { - c} \\ \mathbf{0} \\ {\mathbf{d}} \\ \end{array} } \right) \\ & \forall i \in Irrev: z_{i} = 0 \to v_{Irrev} \le 0 \\ & \forall i \in Rev: z_{i} = 0 \to v_{Rev,i}^{p} = 0 \wedge v_{Rev,i}^{n} = 0 \\ & \left( {1 - z_{i} } \right) \cdot lb_{i} \le r_{i} \le \left( {1 - z_{i} } \right) \cdot ub_{i} \\ & {\mathbf{t}} \in {\mathbb{R}}^{t} ,\quad { }{\mathbf{w}} \in {\mathbb{R}}_{ \ge 0}^{t} ,\quad {\mathbf{v}}_{{{\mathbf{Irrev}}}} \in {\mathbb{R}}_{{}}^{{\left| {Irrev} \right|}} ,\\& {\mathbf{v}}_{{{\mathbf{Rev}}}}^{{\mathbf{p}}} , {\mathbf{v}}_{{{\mathbf{Rev}}}}^{{\mathbf{n}}} \in {\mathbb{R}}_{ \ge 0}^{{\left| {Rev} \right|}} ,\quad {\mathbf{r}} \in {\mathbb{R}}^{{n}} ,\quad { }c > 0,{ }\quad z_{i} \in \left\{ {0,1} \right\} \\ \end{aligned}$$

### Reduced representation of the desired region

The kernel matrix ${\mathbf{K}}$ can also be used to simplify the constraints for the desired behavior in the two MILP variants (14) and (16): the flux vector ${\mathbf{r}}$ of the desired system lies in the nullspace of ${\mathbf{N}}$ and can thus be written as linear combination of columns of the kernel matrix ${\mathbf{r}} = {\mathbf{Ka}}$. For example, by substituting ${\mathbf{r}}$ with ${\mathbf{Ka}}$ in (14), the third row in the matrix disappears and the system reduces to:

17 $$\begin{aligned} & minimize \mathop \sum \limits_{i} z_{i} \\ & s.t. \\ & \left( {\begin{array}{*{20}c} {{\mathbf{N}}^{{\mathbf{T}}} } & {\mathbf{I}} & {{\mathbf{T}}^{{\mathbf{T}}} } & \mathbf{0} \\ \mathbf{0} & \mathbf{0} & {{\mathbf{t}}^{{\mathbf{T}}} } & \mathbf{0} \\ \mathbf{0} & \mathbf{0} & \mathbf{0} & {{\mathbf{D}} \cdot {\mathbf{K}}} \\ \end{array} } \right)\left( {\begin{array}{*{20}c} {\mathbf{u}} \\ {\mathbf{v}} \\ {\mathbf{w}} \\ {\mathbf{a}} \\ \end{array} } \right)\begin{array}{*{20}c} = \\ \le \\ = \\ \le \\ \end{array} \left( {\begin{array}{*{20}c} \mathbf{0} \\ { - c} \\ \mathbf{0} \\ {\mathbf{d}} \\ \end{array} } \right) \\ & \forall i \in Irrev: z_{i} = 0 \to v_{i} \le 0 \\ & \forall i \in Rev: z_{i} = 0 \to v_{i} = 0 \\ & \left( {1 - z_{i} } \right) \cdot lb_{i} \le {\mathbf{K}}_{{{\varvec{i}}, \cdot }} \cdot {\mathbf{a}} \le \left( {1 - z_{i} } \right) \cdot ub_{i} \\ & {\mathbf{u}} \in {\mathbb{R}}^{m} ,\quad { }{\mathbf{t}} \in {\mathbb{R}}^{t} ,\quad { }{\mathbf{w}} \in {\mathbb{R}}_{ \ge 0}^{t} , \quad {\mathbf{v}},{\mathbf{r}} \in {\mathbb{R}}^{{n}} { },\quad { }{\mathbf{a}} \in {\mathbb{R}}^{{{n} - {m}}} ,\quad { }c > 0,{ }\quad z_{i} \in \left\{ {0,1} \right\} \\ \end{aligned}$$

Applying the same transformation to the MILP (16) yields the reduced NB system

18 $$\begin{aligned} & minimize \mathop \sum \limits_{i} z_{i} \\ & s.t. \\ & \\ & \left( {\begin{array}{*{20}c} {{\mathbf{K}}^{{\mathbf{T}}} } & {{\mathbf{K}}^{{\mathbf{T}}} \cdot {\mathbf{T}}^{{\mathbf{T}}} } & \mathbf{0} \\ \mathbf{0} & {{\mathbf{t}}^{{\mathbf{T}}} } & \mathbf{0} \\ \mathbf{0} & \mathbf{0} & {{\mathbf{D}} \cdot {\mathbf{K}}} \\ \end{array} } \right)\left( {\begin{array}{*{20}c} {\mathbf{v}} \\ {\mathbf{w}} \\ {\mathbf{a}} \\ \end{array} } \right)\begin{array}{*{20}c} = \\ \le \\ \le \\ \end{array} \left( {\begin{array}{*{20}c} \mathbf{0} \\ { - c} \\ {\mathbf{d}} \\ \end{array} } \right) \\ & \\ & \forall i \in Irrev: z_{i} = 0 \to v_{i} \le 0 \\ & \forall i \in Rev: z_{i} = 0 \to v_{i} = 0 \\ & \left( {1 - z_{i} } \right) \cdot lb_{i} \le {\mathbf{K}}_{{{\varvec{i}}, \cdot }} \cdot {\mathbf{a}} \le \left( {1 - z_{i} } \right) \cdot ub_{i} \\ & {\mathbf{t}} \in {\mathbb{R}}^{t} ,\quad {\mathbf{w}} \in {\mathbb{R}}_{ \ge 0}^{t} , \quad c > 0,\quad {\mathbf{v}},{\mathbf{r}} \in {\mathbb{R}}^{{n}} ,\quad {\mathbf{a}} \in {\mathbb{R}}^{n - m} ,\quad z_{i} \in \left\{ {0,1} \right\}. \\ \end{aligned}$$

## Results

For benchmarking the different MILP variants, we enumerated MCS in three genome-scale metabolic networks: iJO1366 (*Escherichia coli*), iMM904 (*Saccharomyces cerevisiae*), and iJM658 (*Corynebacterium glutamicum*). As a classical problem for computational strain design, we first computed MCS for the growth-coupled production of a variety of products (iJO1366: 49 products, up to MCS size 7; iMM904: 45 products up to MCS size 7; iJM658: 94 products up to MCS size 8). The calculations were repeated three times with different seeds to induce different branching behavior in the MILP search tree and to thus obtain runtime statistics with higher fidelity. This gives a total of 564 enumerations per setup. As a second class of MCS problems, we also enumerated synthetic lethals in these three networks (up to size 4 in iJO1366 and iMM904, up to size 5 in iJM658). Synthetic lethals are MCS that suppress growth either completely or above some given threshold for the growth rate. Here, 11 thresholds (0.1%, 1%, 10%, …, 90% of the maximal growth rate) were used and the calculations repeated with three different seeds which gives 99 enumerations per setup. Note that computation of synthetic lethals does not involve a desired region and has thus a simpler structure.

With these benchmarks we will compare the following MILP variants for calculating MCS: (1) The main goal of the benchmarks is to compare the runtime behavior of the MILPs derived from (a) the Farkas-lemma-based (FLB) versus (b) the nullspace-based (NB) dual system [Eqs. (14) and (16)]. (2) Afterwards we investigate whether the dimension reduction of the MILP via the kernel-based integration of the desired region in the MILP [Eqs. (17) and (18)] leads to runtime improvements. (3) Finally, it will be tested whether it is advantageous to split reversible dual reaction variables ${\mathbf{v}}$ as done in (14a) and (16a).

During the MCS enumerations, millions of linear programs (LPs) are being solved which, depending on the chosen MILP formulation and type of MCS problem, can be sensitive to numerical issues and sometimes lead to erroneous results. A typical problem that can occur is that an MCS of size $l$ is missed and consequently several supersets of size $l + 1$ are found in the next iteration. However, faulty results of this type are easy to recognize and fix by running additional validation LPs: The correctness of a computed MCS can be checked by running LPs to verify that (i) each MCS is a cut set (must disable the target region), that (ii) it is not a cut set anymore if any knock-out is removed (minimality), and that (iii) the desired region (when given) remains feasible. Apart from erroneous MCS, it may also happen that certain MCS are overlooked. However, as we used different setups and multiple seeds it is reasonable to assume that a result is complete if the same result is being obtained for a variety of conditions. For the pairwise comparison of computation times below we will therefore consider only those calculations which gave the correct results, hence, the number of enumerations that enter a comparison may vary.

All benchmark calculations were performed on a cluster node with two 8-core processors (Intel Xeon Silver 4110) and 192 GB RAM. The different functions for MCS enumeration are part of the latest *CellNetAnalyzer* package [32, 33] (version 2020.2) and the scripts to recalculate the benchmarks can be found on GitHub: (https://github.com/ARB-Lab/FLB_NB_benchmarks).

### Comparing FLB versus NB dual system in the MILP formulation

**Fig. 1:**
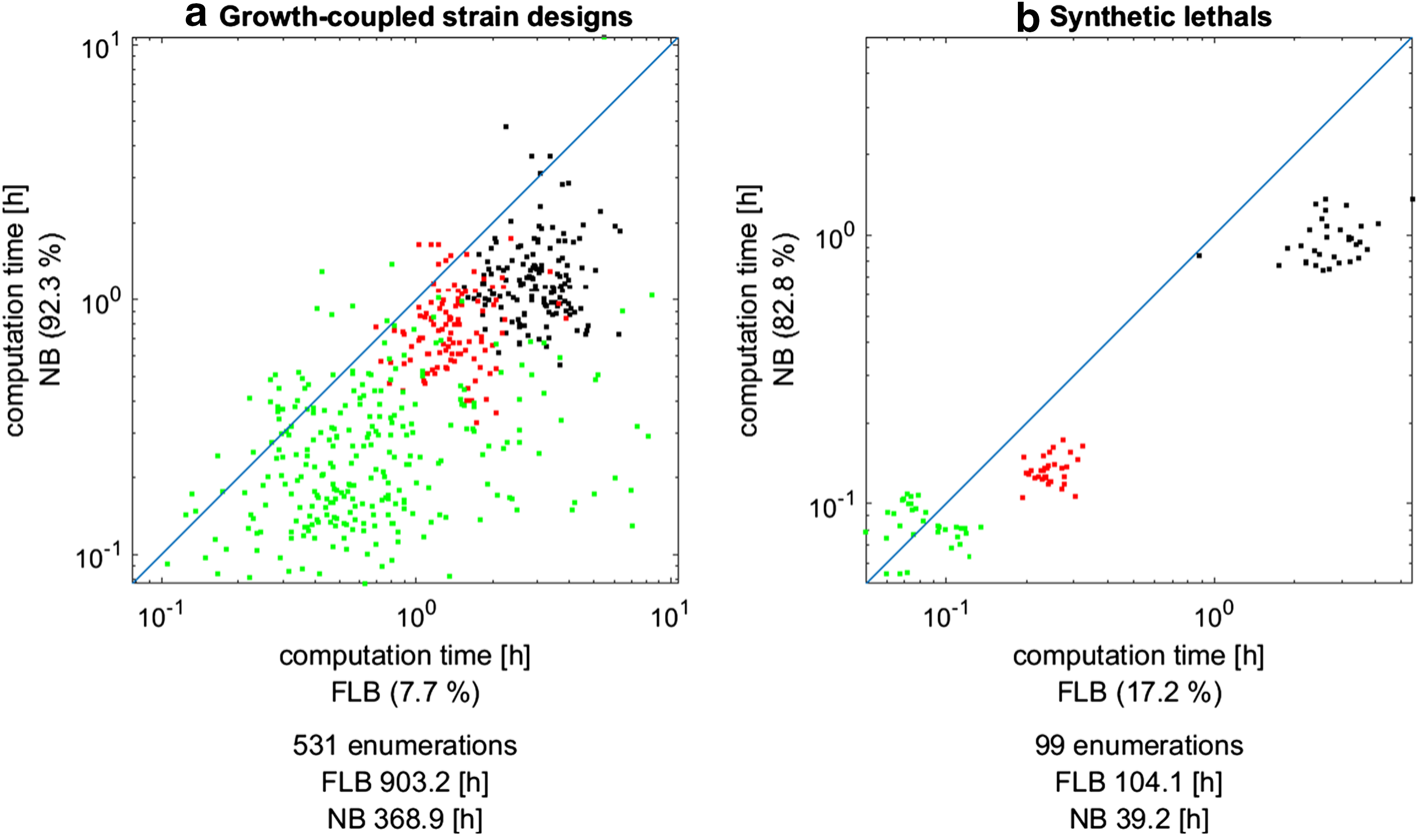
Comparison of computation times of the FLB MILP [Eq. (14)] versus the NB MILP [Eq. (16)] for determining MCS for **a** growth-coupled production strains or **b** synthetic lethals. Each dot represents one particular MCS enumeration scenario (product/organism/seed combination in **a** and growth rate threshold/seed combination in **b**) and the dot color marks the respective model (organism) in which the computation has been conducted: green iJM658; red: iMM904; black: iJO1366. The total number of comparable enumeration scenarios as well as the cumulative sum of the computation times over all scenarios for the MILP variants are given under the diagram. The percentage at the axes quantifies the relative frequency with which the respective MILP variant was faster than the other MILP variant

Figure 1 shows the effect on the runtime when using the NB MILP (16) instead of FLB MILP (14) for enumerating MCS for (a) growth-coupled production (Fig. 1a) and (b) for synthetic lethals (Fig. 1b). The NB variant is in both cases clearly favorable and speeds up the total computation time (over all considered scenarios) by a factor of around 2.5. The advantageous effect can consistently be seen for all three models and both types of problems, although there is a smaller fraction of scenarios (7.7% for growth-coupling MCS, 17.2% for synthetic lethals), where the FLB approach was faster than the NB MILP. However, at least for the synthetic lethals, this pertains to scenarios where the computation time is relatively low.

Beside the computation time one can also evaluate how the two approaches affect the sizes of the actual MILP instances and the memory requirements. These are given in Additional file 1 where it can be seen that the NB MILPs have, as expected, fewer columns (variables) and fewer rows (inequalities) than their FLB counterparts. In contrast, the FLB MILPs have less non-zero entries because stoichiometric matrices are very sparse whereas their kernels typically contain more non-zeros which could, in principle, negatively affect the runtime behavior. The number of binary variables is the same for FLB and NB MILPs, but the latter require a few less indicators. As a result, the memory (peak) requirements appear to be lower for NB MILPs (Additional file 1), although these values have to be taken with caution because CPLEX does not provide comprehensive memory usage logging.

### Effect of using kernel-based description of the desired region

**Fig. 2:**
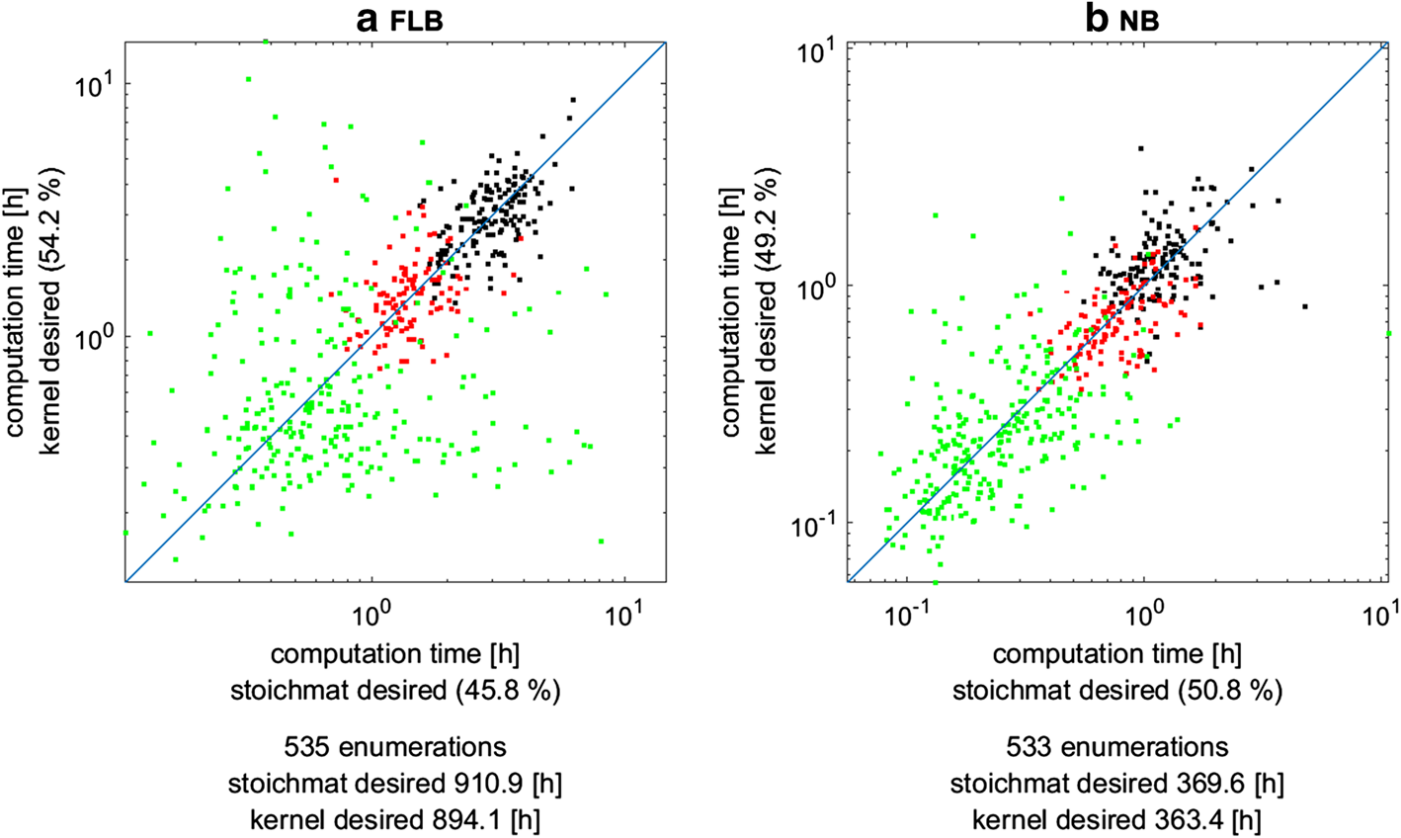
Comparison of computation times of the **a** FLB MILP and of the **b** NB MILP with either the stoichiometric matrix (“stoichmat desired”) or the kernel matrix (“kernel desired”) in the formulation of the desired system. The x-axis and y-axis in **a** refer to the MILPs in Eqs. (14) and (17), respectively, and in **b** to the MILP in Eqs. (16) and (18), respectively. Each dot represents one particular MCS enumeration scenario (product/organism/seed combination) for enumerating MCS for growth-coupled strain designs and the dot color marks the respective model (organism) in which the computation has been conducted: green iJM658; red: iMM904; black: iJO1366. The total number of comparable enumeration scenarios as well as the cumulative sum of the computation times over all scenarios for the MILP variants are given below the diagram. The percentage at the axes quantifies the relative frequency with which the respective MILP variant was faster than the other MILP variant

Next we investigated the effect of using the kernel (instead of the stoichiometric) matrix for expressing the constraints of the desired region. We tested this for both MILP variants, first for the FLB dual system [MILP (14) against MILP (17); Fig. 2a] and then for the NB dual system [MILP (16) against MILP (18); Fig. 2b]. Since synthetic lethals do not involve desired regions, these benchmarks were restricted to MCS for growth-coupled strain designs. Despite its reduced dimension, the kernel-based formulation of the desired system led to only marginal overall improvements (~ 1.5%) in both MILP variants. For the FLB approach, there was even a slightly higher number of cases where the variant with the stoichiometric matrix in the desired system was faster (although the cumulative computation time over all scenarios was higher).

### Effect of separating irreversible and reversible reactions

The FLB MILP formulation in Eq. (14) is more condensed than previously used variants because it (1) uses a reduced and non-redundant formulation of indicator constraints, (2) uses only one binary variable $z_{i}$ per dual reaction variable $v_{i}$ and (3) does not separate and split dual reaction variables $v_{i}$ related to reversible reactions. In dedicated benchmarks we found that (1) and (2) indeed speed up the calculations in both FLB and NB MILP with a factor of 1.2–4 (not shown). Regarding the third simplification, in a last set of benchmarks we compared the condensed representations of the FLB (14) and NB (16) MILP representations against the equivalent but expanded formulations given in (14a) and (16a), where dual variables $v_{i}$ related to reversible reactions have been split.

**Fig. 3:**
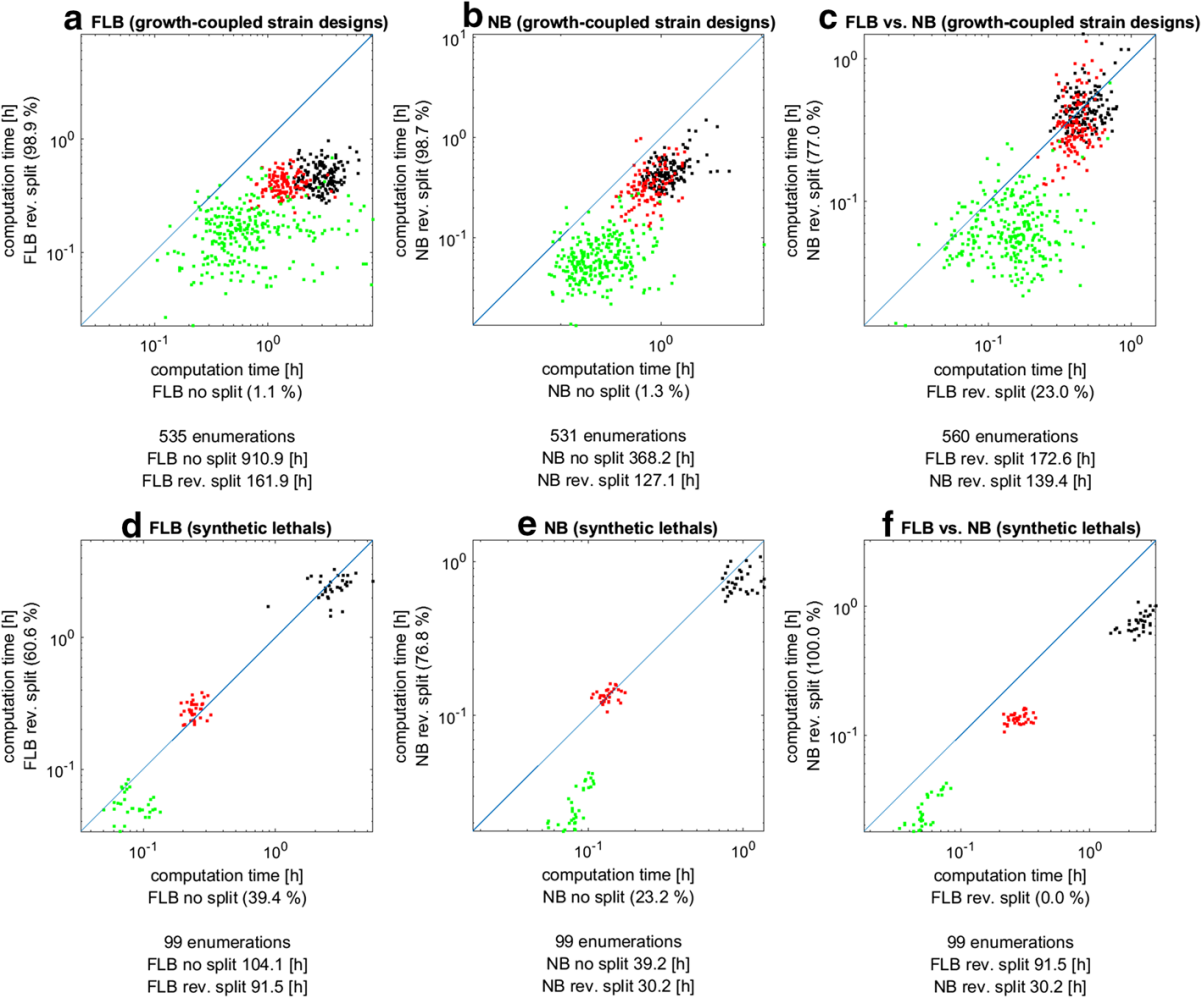
Comparison of computation times of the FLB MILP and the NB MILP when used either with the condensed (“no split”) MILP variant [Eqs. (14) and (16), respectively] or the (“rev. split”) MILP variant with separated and split reversible reactions [Eqs. (14a) and (16a), respectively]. **a**, **d** “no split” versus “rev. split” in FLB for MCS for growth coupling (**a**) and for synthetic lethals (**d**); **b**, **e** “no split” versus “rev. split” in NB for MCS for growth coupling (**b**) and for synthetic lethals (**e**); **c**, **f**: comparison of “rev. split” variants for FLB and NB for MCS for growth coupling (**c**) and for synthetic lethals (**f**). Each dot represents one particular MCS enumeration scenario (product/organism/seed combination in **a**–**c** and growth rate threshold/seed combination in **d**, **e** and the dot color marks the respective model (organism) in which the computation has been conducted: green iJM658; red: iMM904; black: iJO1366. The total number of comparable enumeration scenarios as well as the cumulative sum of the computation times over all scenarios for the MILP variants are given below the diagram. The percentage at the axes quantifies the relative frequency with which the respective MILP variant was faster than the other MILP variant

Surprisingly, although the number of variables increases (for the reversible reactions, the number of $v_{i}$ variables is doubled), splitting the $v$ variables has a strong beneficial effect in both FLB and NB MILP (Fig. 3). In particular, for computing MCS representing growth-coupled strain designs, the runtime for the FLB MILP improves by a factor of 5.6. For the new NB MILP, the speed-up factor is 2.9 and thus still significant, however, due to the smaller relative improvement, the overall advantage of the NB over FLB is reduced from a factor of 2.5 (Fig. 1a) to 1.24 (Fig. 3c). Less significant but still relevant speed-ups of the split MILP variants can be observed when computing MCS representing synthetic lethals, where the runtimes are improved by 15% (FLB; Fig. 3c) and 30% (Fig. 3e). As the split variant of the NB MILP improves relatively better than the FLB MILP, the former even expands its advantage over the FLB variant in this case (averaged speed-up factor of 3.0 for NB; Fig. 3f).

## Discussion

The development of a duality-based algorithmic framework for MCS computation was a cornerstone to allow the enumeration of thousands of MCS in genome-scale networks via mixed-integer linear programming. Several recent refinements and extensions contributed to the improved performance of the respective algorithms and broadened the spectrum of applications in systems biology and computer-aided metabolic engineering.

In this work we introduced several modifications and variants of the duality-based core algorithm and benchmarked their effects on the overall performance. The main theoretical results build upon the recently introduced MCS^2^ approach [21] and extend it in several directions. In contrast to the Farkas-lemma-based dual system used in earlier studies, the MCS^2^ approach employs a more condensed representation of the dual system based on the nullspace of the stoichiometric matrix, which, due to its reduced dimension, holds promises to further enhance MCS computations. Herein, we generalized the original NB approach (which was limited to blocking the operation of certain target reactions) to the most general case of MCS computations with arbitrary target and desired regions. Moreover, we introduced a new MILP variant which fully leverages the reduced size of the NB dual system. With a large set of benchmarks we could show that this NB MILP outperforms the MILP based on the FLB dual system speeding up MCS computation with an averaged factor of approximately 2.5. Thus, in contrast to the findings in [21], with our version of the NB MILP we could demonstrate a significant benefit compared to the conventional FLB MILP. Although the latter has the same number of binary (indicator) variables as the NB MILP (which is often seen as a main factor for the complexity of a MILP), the reduced number of continuous variables ($m$ less) and equalites ($m$ less; see Table 1) leads in most cases to a decrease of the runtime and peak memory usage. The new NB MILP thus seems to be the fastest currently known algorithm for the dual calculation of MCS. However, it should be noted that there is no guarantee that the NB MILP is always faster than the FLB MILP for a given MCS problem. For example, despite the averaged speed-up factor of 2.5, there were a few cases in all three considered networks where the FLB MILP ran faster than the NB version (see Fig. 1a). Furthermore, although the use of the kernel instead of the stoichiometric matrix in the dual reduces the dimension of the NB MILP, the kernel matrix has usually more non-zero entries than the normally very sparse stoichiometric matrix (Additional file 1), which could potentially lead to adverse effects when using the NB MILP. This might also be the reason why the replacement of the stoichiometric matrix with the kernel matrix in the description of the desired system in the FLB MILP (17) and NB MILP (18) led only to minor runtime improvements. Methods that help to compute sparse kernel matrices might therefore help to further boost the NB MILP (and the FLB MILP with a kernel-based formulation of the desired system).

Apart from the generalized NB dual system and its MILP, we also introduced simplifications for the formulation of constraints related to binary MILP variables. These simplifications can be applied in both FLB as well as NB MILPs and allow a very compact representation of MCS-related MILPs [Eqs. (14) and (16)], despite of the wide range of problems that can be addressed with them. We found that most of these condensed formulations accelerated the respective calculations, however, the number of variables and constraints of two different MILP variants is not always a predictor for their relative performance as was shown in the third set of our benchmarks. Separating and then splitting the dual variable of reversible reactions in the FLB MILP (14a) and in the NB MILP (16a) increases the number of continuous variables but it nevertheless led to a remarkable up to fivefold speed-up. This example also shows that the relative outperformance of the NB over the FLB MILP may vary with (seemingly minor) modifications in the respective MILP formulation and also with the type of MCS problem (e.g., MCS for growth-coupled strain designs vs. synthetic lethals).

We finally note that the new MCS MILP variants presented herein can be easily adapted to generalized MCS problems recently proposed in [24], including the consideration of multiple target and desired regions or the computation of gene MCS.

## Conclusion

In this study we presented several algorithmic advancements for the dual calculation of MCS in metabolic networks. This includes (1) a generalization of the recently introduced nullspace-based dual network approach, (2) its translation to a corresponding MILP with reduced dimensions compared to previous configurations, and (3) several simplifications in the formulation of constraints related to binary MILP variables. A large set of benchmark calculations was performed, demonstrating the superior performance of the new nullspace-based MILP formulation but also revealing that highly condensed formulations of constraints, especially on reversible reactions, may behave worse than variants with a larger number of (more explicit) constraints and involved variables. Our results are of high importance for theoretical and algorithmic developments as well as for practical applications of the MCS framework.

## Supplementary information

**Additional file 1.** Tables with information on MILP sizes and average peak memory requirements for the different computations shown in Figs. 1–3.

## Data Availability

A GitHub repository containing all functions and scripts to run the benchmarks are available at: https://github.com/ARB-Lab/FLB_NB_benchmarks. Operating system(s): Cross-platform. Programming language: Matlab. Other requirements: IBM^®^ ILOG^®^ CPLEX^®^ Optimization Studio, *CellNetAnalyzer* version 2020.2 or higher. License: Apache License, Version 2. Any restrictions to use by non-academics: none.

## Abbreviations

MCS: Minimal cut sets
EM: Elementary modes
FLB: Farkas-lemma-based
NB: Nullspace-based
MILP: Mixed integer linear program
